# Estimation of Transcription Factor Activity in Knockdown Studies

**DOI:** 10.1038/s41598-019-46053-7

**Published:** 2019-07-03

**Authors:** Saskia Trescher, Ulf Leser

**Affiliations:** Knowledge Management in Bioinformatics, Computer Science Department, Humboldt-Universität zu Berlin, Unter den Linden 6, 10099 Berlin, Germany

**Keywords:** Computational models, Gene regulation, Gene regulatory networks

## Abstract

Numerous methods have been developed trying to infer actual regulatory events in a sample. A prominent class of methods model genome-wide gene expression as linear equations derived from a transcription factor (TF) – gene network and optimizes parameters to fit the measured expression intensities. We apply four such methods on experiments with a TF-knockdown (KD) in human and *E. coli*. The transcriptome data provides clear expression signals and thus represents an extremely favorable test setting. The methods estimate activity changes of all TFs, which we expect to be highest in the KD TF. However, only in 15 out of 54 cases, the KD TFs ranked in the top 5%. We show that this poor overall performance cannot be attributed to a low effectiveness of the knockdown or the specific regulatory network provided as background knowledge. Further, the ranks of regulators related to the KD TF by the network or pathway are not significantly different from a random selection. In general, the result overlaps of different methods are small, indicating that they draw very different conclusions when presented with the same, presumably simple, inference problem. These results show that the investigated methods cannot yield robust TF activity estimates in knockdown schemes.

## Introduction

The regulation of gene expression is a fundamental biological mechanism in all living species. It determines the cells’ unique properties and enables them to adapt to the organism’s development, cellular function, the environment and external stimuli^1^. Gene regulation also plays an important role in the development and progression of various diseases^2,3^. Thus, the elucidation of human regulatory relationships is an important research field and many methods attempting to infer the actual regulatory events in a given sample have been proposed.

In eukaryotes, gene expression is mainly regulated by a complex network of transcription factors (TFs) which activate or repress gene transcription^1^. Several algorithms, such as biRte^4^ (Bayesian inference of context-specific regulator activities), ISMARA^5^ (Integrated System for Motif Activity Response Analysis), RABIT^6^ (Regression Analysis with Background Integration) and RACER^7^ (Regression Analysis of Combined Expression Regulation), have been presented to model genome-wide gene expression and regulation via the activity and relationships of transcription factors. These models allow for the application of mathematical optimization to find parameters that minimize the divergence of predicted and measured expression intensities^8^. They all consider the topology of the regulatory TF – gene network to be given and try to infer the actual TF activity developed in a certain disease or under a specific experimental condition. Their primary output is a ranked list of TFs, sorted by their activity in a given group of samples. A detailed description of the specific methods evaluated in this paper is given in the materials and methods section at the end of the paper. Several studies reported that such methods can be used to identify biomarkers for specific phenotypes in human cell lines and *in vivo* samples, for example in innate immunity^5^, ageing related changes^5^ or acute myeloid leukemia^7^.

We previously compared different methods for estimating regulatory activity qualitatively and quantitatively in detail^8^. We used a publicly available human TF – gene network^9^ together with experimental data from TCGA^10^ (The Cancer Genome Atlas) for three cancer types to identify key biomarkers for these specific diseases. The results showed that all methods seemed to detect strong signals and find biologically relevant information, but sensitivity was low and the mutual result overlaps from different methods were small. We suspected that the complexity of gene regulation in cancer was one reason for the questionable performance and low consistency of different methods’ results. Therefore, we here focus on much less complex data and use knockdown experiments to evaluate different methods on estimating TF activity changes. We suppose that the highest change in activity will occur in the knocked down TF when comparing case and control samples. Many datasets of such high-throughput experiments for certain experimental conditions and different species have been published and are available in public repositories like GEO^11^ (Gene Expression Omnibus). In this straightforward and for the methods favorable setting, we expected that the methods would consistently be able to identify the knocked down TF.

**Figure 1:**
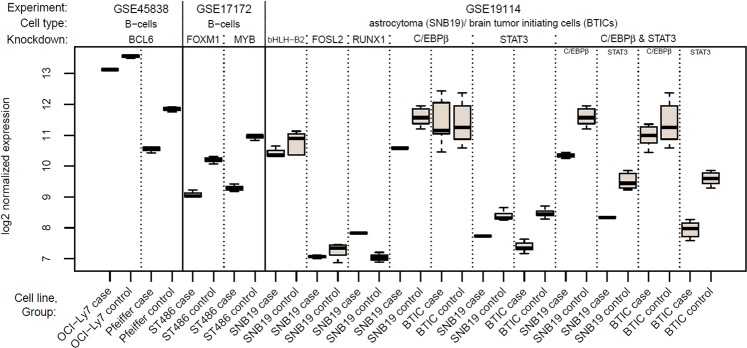
Boxplots of log2 normalized expression values for all human KD TFs, comparing respective case and control groups. For the double KD *C/EBPβ & STAT3*, separate boxplots for both TFs are shown. In all experiments, expression in case samples is significantly lower than in control samples, except for *C/EBPβ* (single and double KD) in BTICs and *RUNX1* KD.

**Figure 2:**
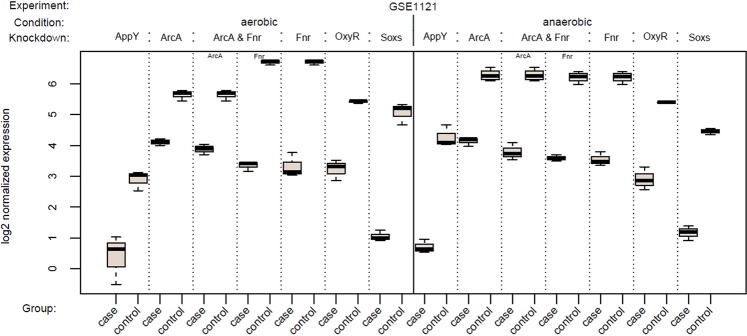
Boxplots of log2 normalized expression values for all *E. coli* KD TFs, comparing respective case and control groups. For the double KD *ArcA & Fnr*, separate boxplots for both TFs are shown.

**Figure 3:**
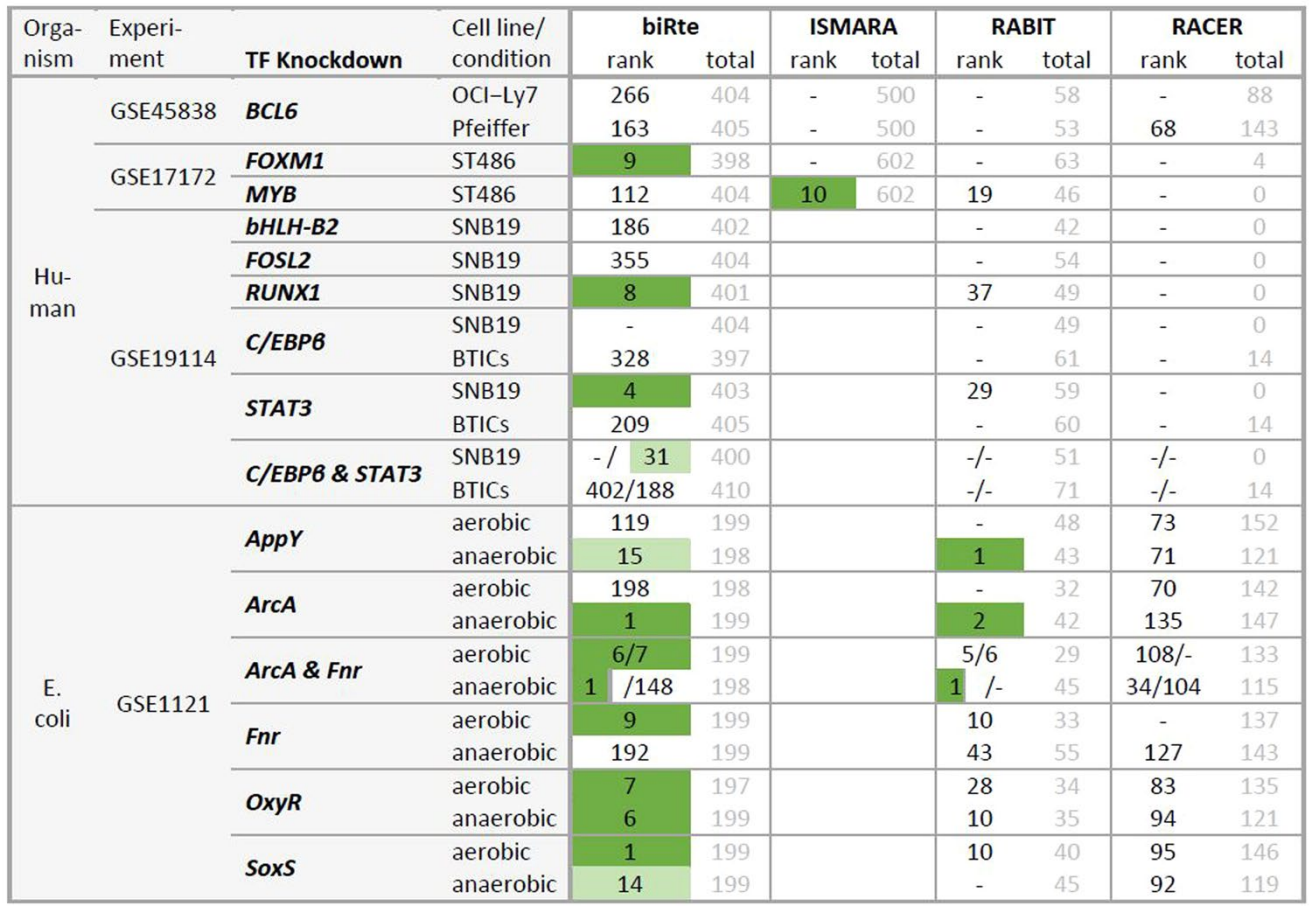
Ranks of knocked down TFs and total number of ranked TFs per method and data set. Ranks in the top 5% of all ranked TFs are marked in green and ranks in the top 5–10% in light green. Two ranks in one table cell refer to a combined knockdown of two TFs and are given in the order of the TFs at the beginning of the table row. An empty table cell (in ISMARA column) indicates that the method was not applicable to the data set. A dash is shown when a TF was not ranked by a method (see text for explanation of different numbers of ranked genes).

Here, we compare four different methods, namely biRte^4^, ISMARA^5^, RABIT^6^ and RACER^7^, to infer transcription factor activity from gene expression data in knockdown (KD) experiments. We downloaded transcriptome data of four publically available KD experiments from the GEO^11^ repository including different TF knockdowns in human and *E. coli* cell lines. To better distinguish KD TFs from cell lines or other abbreviations, we will set TFs in italics. The three chosen experiments in human cell lines (GEO identifier GSE45838^12^, GSE17172^13,14^ and GSE19114^15^) contain data from 8 knocked down genes (*BCL6*, *FOXM1*, *MYB*, *bHLH-B2*, *FOSL2*, *RUNX1*, *C/EBPβ*, *STAT3*) and the double knockdown *C/EBPβ & STAT3*. The selected experiment in *E. coli* (GEO identifier GSE1121^16^) comprises 5 knocked down genes (*AppY*, *ArcA*, *Fnr*, *OxyR*, *SoxS*) and the double knockdown *ArcA & Fnr*. Some of these experiments were conducted in different cell lines or conditions (see Materials and Methods, section Transcriptome Data). Overall, we study 25 data sets (combinations of the experiment, the particular TF knockdown and different cell lines or growth conditions), 13 from human and 12 from *E. coli*. For an overview of the composition of the experiments, see Figs 1, 2 and 3. Throughout the paper, we refer to the whole KD experiments from GEO as “experiments”, which contain different KDs in cell lines or growth conditions, called “data sets”. Together with the transcriptome data, we used two gene regulatory networks (one for human, one for *E. coli*) as input to the methods biRte, RABIT and RACER, whereas ISMARA employs an own, inaccessible underlying network. The network including information on human regulatory relationships is based on a text-mining approach^9^ complemented with TF – gene interactions from the public TRANSFAC database^17^. The network was built by text mining the entire Medline and an additional manual curation step of the top-ranking sentences. It thereby combines the content of regulatory databases with more than 300 validated regulatory relationships. The network for *E. coli* was retrieved from RegulonDB^18^. The considered datasets are described in detail in the materials and methods section at the end of the paper.

We predominantly assessed the rank of the TF that was knocked down and the total number of ranked TFs. We additionally checked for aliases and we determined the ranks of neighbor TFs in the network, of co-members in a pathway and of interacting TFs. To examine whether the methods were able to detect a common signal in the data, we compared the overlap of the top 100 ranked regulators of all methods within one data set. We additionally performed the activity estimation on smaller networks and on networks inferred de-novo by ARACNE^19^ (Algorithm for the Reconstruction of Accurate Cellular Networks) to assess the influence of the network on the results. To test whether the mere differential expression is a better predictor for TF activity changes, we calculated ranks of differential expression for the knocked down TFs and compared those to the activity ranks.

Apart from our own previous work^8^, we are not aware of any other independent study on the performance of optimization-based algorithms for the estimation of whole genome transcription factor activity. Our previous results compared the performance on multi-omics data sets where no clear expectations could be formulated and also found very low coherence in the results of different methods. This lack of quality estimation of results led us to the new experimental design of using knock-down. However, also on this presumable much simpler problem the result overlaps are very low and the knocked-down transcription factor was only very rarely identified. We conclude that the investigated methods do not yield robust estimates of TF activity in a knockdown scheme.

## Results

### Overview

Our results show that, although almost all KD TFs showed differential expression, their activity ranks were only in 15 out of 54 cases within the top 5% of all ranked TFs (compare Fig. 3). In *E. coli*, the identification of the KD TF by activity estimation yielded slightly better results compared to human cell lines. When looking not only at the KD TF but at the regulators related to the KD TF in the network or a pathway, we identified only a single case where the mean of the ranks of all related TFs was significantly smaller than expected by chance. The overlap of the top 100 ranked regulators of all methods within one data set was small and statistically insignificant. The reduction of the network size or the use of ARACNE’s inferred networks did not improve the results.

### Differential expression of knocked down TFs

First, we tested whether the knocked down (KD) TFs themselves were differentially expressed, which was the case for all human KD TFs except *C/EBPβ* in BTICs (brain tumor initiating cells), both in the single KD and double KD together with *STAT3*, see Fig. 1. Unexpectedly, the expression of *RUNX1* was significantly upregulated in SNB19 case samples compared to the control samples. Nonetheless, we included *RUNX1* in our analyses since we were only interested in finding absolute TF activity changes. In *E. coli* (see Fig. 2), all KD TFs were significantly downregulated in the corresponding case samples. The according p-values are given in Supplementary Table S1.

Additionally, we checked whether the differential expression per se would be a good predictor for the determination of knocked down TFs in a data set. Therefore, we computed differential expression separately in each data set, contrasting the expression of the corresponding case and control samples and evaluated the ranks of differential expression for the KD TFs. The results are shown in Supplementary Fig. S1. In human, in 9 out of the 13 data sets, TFs were ranked within the top 5%. In *E. coli*, the number of KD TFs in the top 5% TFs was 3 in the aerobic and 5 in the anaerobic condition out of 6 data sets in either condition. As expected, the KD TFs are in about two third of the considered data sets amongst the TFs with the highest changes in differential expression.

### Ranking of knocked down TFs

We next applied biRte, ISMARA, RABIT and RACER to determine the respective KD TFs’ ranks. The KD TFs were only in 15 out of 54 cases within the top 5% of all ranked TFs (4 out of 18 in human and 11 out of 36 in *E. coli*). Of the 54 cases, where ranks were provided, 27 resulted from biRte, one from ISMARA, 13 from RABIT and 13 from RACER. Due to stringent filtering thresholds within the methods, no activity score was assigned to the KD TF in 37 cases, hence the ranking could not be computed in those cases.

The resulting ranks of knocked down TFs and the total number of ranked TFs per method and data set are shown in Fig. 3. Favorable results, meaning that the knocked TF was highly ranked, are marked in green.

We observed that biRte provided ranks for nearly all KD TFs. In 3 out of the 13 human data sets, where ranks were specified, biRte ranked the knocked down TF in the top 5% (*FOXM1*, *RUNX1* and *STAT3* in SNB19). In *E. coli*, the results from biRte were better with 8 out of 14 TFs in the top 5% and another two TFs in the top 10%. In all other data sets, the ranks provided by biRte for the TFs in question were quite low. ISMARA could only be applied to GSE45838 and GSE17172 since the chips from the other experiments were not supported by the online interface. In one data set (*MYB*) the KD TF was highly ranked (10^th^ out of 602), but ISMARA did not provide any ranks for the two other KD TFs (*BCL6* and *FOXM1*). Since the underlying network from ISMARA is not accessible we cannot discern whether the TF is not present in the network or was not considered important by the ranking procedure. RABIT removes TFs with insignificant cross-sample correlation from the results and therefore only provides the ranks of, on average, 56 TFs in our analyses. It did not provide any activity score for the KD TF in over half of the data sets (12 in human, 4 in *E. coli*). In human, not a single KD TF was ranked in the top 20%. However, in *E. coli* RABIT was able to identify *AppY* (rank 1) and *ArcA* as knocked down TFs in the anaerobic condition (rank 2 in the single KD and rank 1 in the combined KD *ArcA & Fnr*). In contrast, RACER ranked only one KD TF for the human data sets at all (*BCL6*) and did not rank any KD TF highly in *E. coli*. In some human data sets, RACER even reported the total number of important regulators to be zero.

### Ranking of related TFs

We expected that the knockdown of a certain TF should not only affect the activity of this TF itself, but also influence the activity of related TFs. Therefore, for each KD TF, we determined the ranks of a set of related regulators. We defined as related all TFs directly connected in the same pathway (information from SignaLink^20^ for human respectively EcoCyc^21^ for *E. coli*), direct neighbors in the TF – gene network, directly interacting TFs (information from TcoF-DB v2^22^, human) and presumed aliases from the GeneCards^23^ (human) and EcoCyc^21^ database (*E. coli*) (see Materials and Methods for the procedure and Supplementary Table S2 for the collection).

**Figure 4:**
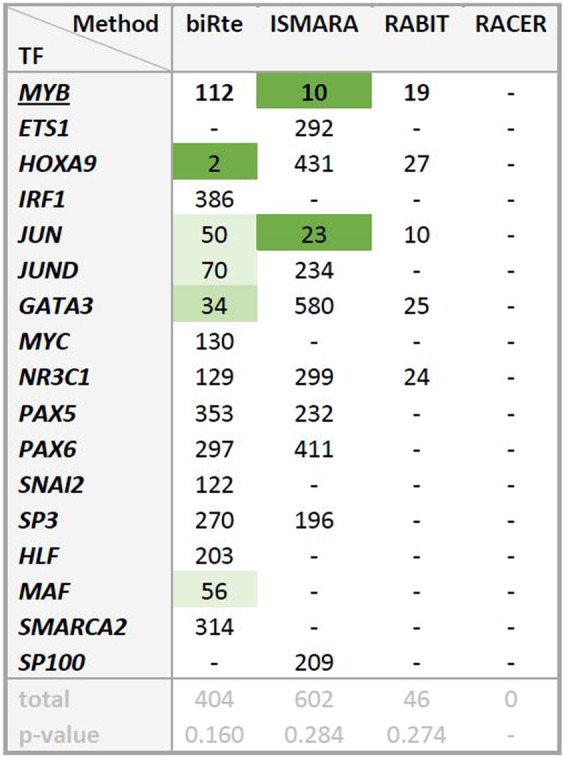
For experiment GSE17172: Ranks of *MYB* (bold) and related TFs, total number of ranked TFs per method and p-value indicating significance of test whether the mean of the ranks of all related TFs is smaller than the average rank. Ranks of TFs in the top 5% of all ranked TFs are marked in dark green, ranks in the top 5–10% in green and ranks in the top 10–20% in light green. When a TF was not ranked, “−” is shown.

We show the resulting ranks and according p-values of the KD TF and related TFs for one exemplary result (*MYB* KD from GSE17172) in Fig. 4, all other results are given in Supplementary Fig. S2 Again, we observed that also the related TFs are rarely ranked highly by any of the methods. Only one related TF (*JUN*), which is directly connected to *MYB* in the human regulatory network, was ranked among the top 20% TFs by two of the four methods (biRte: rank 50, ISMARA: rank 23). Previously, it was shown that *JUN* contributed to the transcriptional activation of *MYB*^24,25^. For each method and data set individually, we evaluated whether the mean of the resulting ranks of all related TFs was significantly smaller than the average rank expected at random (total number of ranked TFs divided by 2). Only one out of 54 of the mean ranks of the estimated activity changes was significantly below the average rank: in *E. coli*, biRte ranked *OxyR* and a related TF highly in the anaerobic condition (p = 0.002). However, since this result was obtained with quite a small sample set (only two ranked TFs), we consider it not representative.

### Overlap of top 100 TFs

Since the ranks of knocked down and related TFs were quite different in each method, we examined whether the methods might detect a common signal in the data such as a drastic change elsewhere in the network incurred by the KD. To this end, we compared the overlap of the top 100 ranked regulators of all methods within one data set.

We found very little overlap in human cell line data. The highest overlap among three methods (biRte, RABIT and ISMARA) occurred in *FOXM1* with only four common TFs within the top 100 (*JUN*, *MYBL2*, *NR2F2* and *FOXO4*). These results make sense, as the expression of *FOXM1* and *MYBL2* as its downstream factor were significantly associated with clinical stages and overall survival of glioma patients^26^ and is very high in Burkitt lymphoma^27^. Further, *MYBL2* deregulations occurred in a broad spectrum of cancer entities^28,29^. *FOXM1* is a direct target of repression by *FOXO* proteins. An inactivation of *FOXO* or overexpression of *FOXM1* was associated with tumorigenesis and cancer progression^30^. Nevertheless, the overlap is extremely small and not significantly larger than expected at random (p-value = 0.81, tested by simulating the size of the overlap of three lists when sampling 100.000 times 100 out of 429 TFs per list).

**Figure 5:**
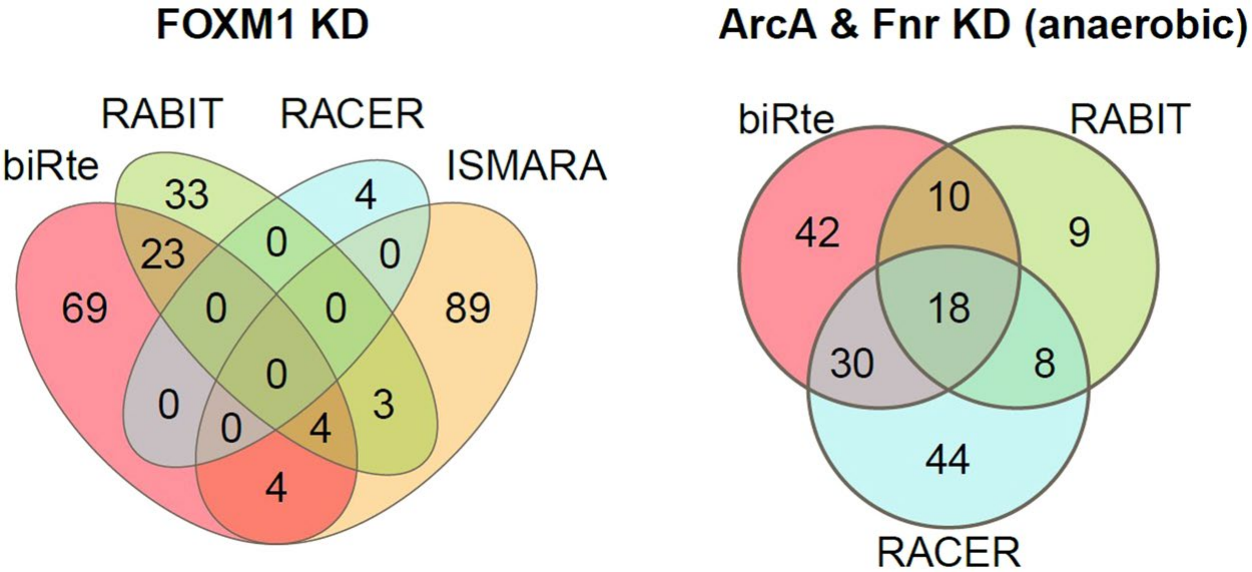
Number of overlapping TFs in the top 100 by estimating TF activity with different methods. Venn diagrams are shown for *FOXM1* knockdown in human (left) and for the combined *ArcA & Fnr* knockdown in *E. coli* for the anaerobic condition (right). For RABIT and RACER, the total number of ranked TFs was below 100 in some cases (see Fig. 3).

In *E. coli*, the number of common TFs from biRte, RABIT and RACER was higher, but also not significant (p-value = 0.96), with a maximum overlap of 18 TFs (*ArcA & Fnr* knockdown in the anaerobic condition). The overlap contained, for example, *ArcA*, which is activated in anaerobic conditions^31^, *NtrC*, which was shown to be upregulated during the transition from anaerobic to aerobic conditions^32^, and *AdiY*, which was maximally induced under anaerobic conditions^33^. Although the methods do not find the knocked down TF itself, at least in our *E. coli* datasets they commonly find TFs biologically relevant for the condition under consideration. The results are exemplarily shown for *FOXM1* and the combined *ArcA & Fnr* KD (anaerobic condition) in Fig. 5 and in Supplementary Fig. S3 for all other TFs.

### Network alterations

**Figure 6:**
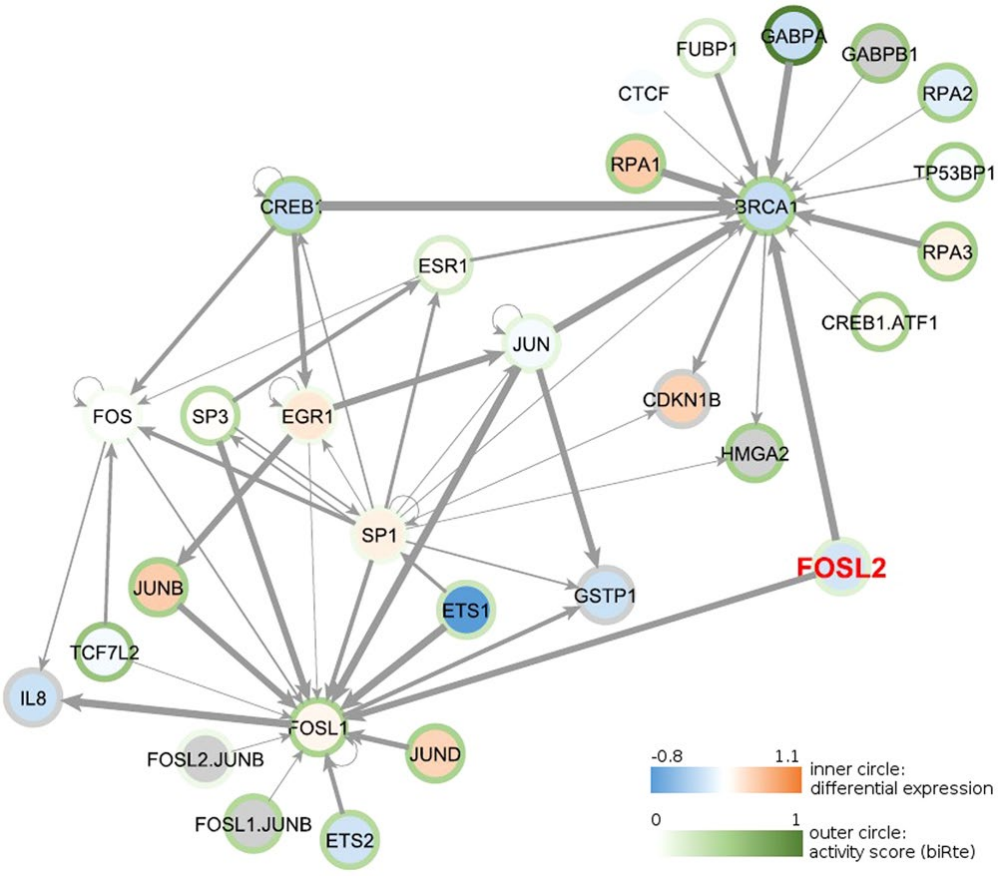
Restricted network for *FOSL2*. The color of the inner circle corresponds to the differential expression of case vs control samples from GSE19114, SNB19 cell line with *FOSL2* knockdown (log2 fold changes): blue colors correspond to downregulated, red colors to upregulated genes in the case samples; genes with missing expression are colored in grey. The color of the outer circle corresponds to the inferred activity score from biRte, ranging from 0 (no activity, white) to 1 (high activity, dark green). The edge width corresponds to the absolute correlation of the expression values between the two adjacent nodes: small absolute correlation values are marked with a thin line, higher absolute correlation values with bolder lines. Edges with missing correlation values and self-correlation were given the thinnest line width.

**Figure 7:**
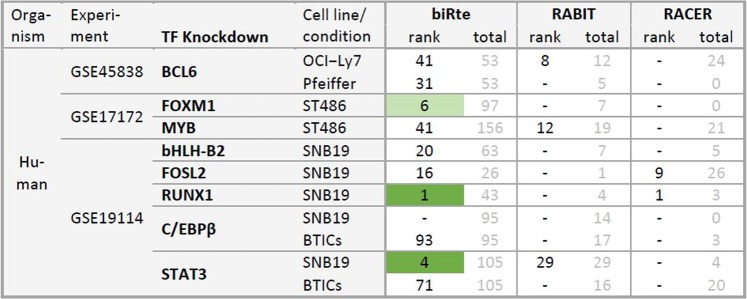
Ranks of KD TFs and total number of ranked TFs per method and data set for the restricted networks. Ranks of KD TFs in the top 5% of all ranked TFs are marked in green and ranks in the top 5–10% in light green. When a TF was not ranked, “−” is shown.

The previous results showed that, in a few cases, the methods were able to find biologically plausible information, although they did not identify the knocked down TF or its functional vicinity. One possible reason for this observation, which is in contrast to results published with the methods^4,6,7^, is that the regulatory networks used in the original work were much smaller compared to our networks. To assess whether the usage of a smaller network improves the results, we restricted the underlying TF – gene network to the neighborhood of each knocked down TF with a distance of two. Note that this design gives a very favorable prior to the analysis. An exemplary restricted network for *FOSL2* is presented in Fig. 6. We applied biRte, RABIT and RACER again using these individual smaller networks for the human data sets and performed TF ranking. The resulting TF activities are shown in Fig. 7 and are not better than for the full networks. Only *RUNX1* and *STAT3* were ranked within the top 5% and *FOXM1* in the top 10% using biRte. This result was already obtained using the full network (compare Fig. 3). We conclude that the use of smaller and more focused regulatory networks alone is not sufficient to obtain more accurate results in human.

To further study the influence of the underlying regulatory network, we applied the popular method ARACNE^19^ to reconstruct ab initio a gene regulatory network from the given transcriptome data exemplarily for the *FOXM1* KD (human) and *AppY* KD (*E. coli*). We used these networks as input to the investigated TF activity estimation methods and ranked the resulting TF activity scores. Although the networks inferred by ARACNE have a higher density compared to our original networks, the resulting TF activity rankings are comparable (see Supplementary Fig. S4). Therefore, the network provided as background knowledge to the methods seems not to be the most important element to explain the overall bad performance.

## Discussion

We conducted a comparative evaluation of different transcriptome-based TF activity estimation methods using knockdown data sets. Our results are easily reproducible since they are based on publically available data sets, networks and methods. The results show that the investigated methods do not yield robust estimates of TF activity in a knockdown scheme: Only in around a fourth of all cases the KD TF was ranked within the top 5%, and in many cases, the methods did not provide any score for the KD TF due to the internal filtering.

This poor overall performance cannot be attributed to a low effectiveness of the knockdown, which had an enormous effect on the TF’s gene expression: Nearly all KD TFs showed a significantly high differential expression and most of them had one of the highest changes in differential expression of all genes in the respective data set. We expected the methods to recognize such a drastic change in expression and activity represented by the KD. However, they could only rarely find the KD TF even when its differential expression was very high. This might indicate, that the KD itself affects only a small portion of the whole gene expression. Then one could argue, that the methods do not detect such particular changes and seem to be robust against limited variation in the input data. Nonetheless, the KD signal was clearly present in the data and expected to be found by the methods.

Also, the network size is not a negative factor for prediction performance, since the use of smaller networks did not improve the detection of KD TFs for any of the activity estimation methods. When using other underlying regulatory networks, like the ones provided by ARACNE, the resulting TF activity rankings were not different from previous results. Therefore we conclude that the results are not completely imposed by the network given as input to the methods. We can also exclude the number of samples within a data set as a restricting element, as data sets with more samples did not achieve better results than those with fewer samples.

Overall, the results from *E. coli* are better compared to the results from human cell lines, both regarding the detection of the KD TF and regarding the agreement among different methods. The gene regulatory network of *E. coli* is probably the best characterized one of all species^34^ with a gold standard of experimentally validated interactions from RegulonDB^18^. Even under such optimal conditions, the obtained results have only a poor quality. Conversely, a comprehensive characterization of the human regulatory repertoire is lacking since only about half of the estimated 1,500–2,000 TFs in the mammalian genome is known^35^ and the existing knowledge about regulatory effects is scattered over the biological literature and different, partly commercial, databases, impeding the construction of comprehensive networks^9^. We expected that the estimation of TF activity in human is a much harder task compared to its estimation in *E. coli*, which is partly confirmed by our results.

Even in *E. coli*, some methods were not able to detect the knocked down TF, only biRte yielded moderate results. RABIT and RACER discarded too many TFs before the scoring step due to an insignificant cross-sample correlation, indicating that the model and feature selection procedures might be too stringent. In some of the experiments we chose from GEO^11^, the sample size was relatively small with on average 4 case and 6 control samples per data set and a partly high variation within the groups (compare Figs 1 and 2). However, even in the datasets with larger sample size or with smaller variation, the method’s results were not better compared to the less favorable datasets.

We further observed that related TFs were rarely ranked highly by any of the methods and their ranks did not differ significantly from a random set. Therefore, we examined whether the methods were able to detect a common signal in the data at all and compared the overlap of the top 100 ranked regulators of all methods within one data set. The overlap in human data was quite small, but consistently larger in *E. coli*. We attribute the low similarity of the results partly to the noisy character of the transcriptome data provided as input. Furthermore, gene regulation has an inherent time dependency which is neither covered by the experimental setup nor by the mathematical models. Also, many other factors important for regulation, like chromatin structure or posttranscriptional effects, are ignored. Therefore, finding a knocked down TF seems to be a difficult problem to the examined methods. However, in both human and *E. coli*, the intersection of methods identified some biologically plausible TFs for the condition under consideration. In the literature, we found many examples of such evaluation procedures^4–7^, where highly ranked TFs were found to be biologically important.

For the evaluation of the experiments based on human data, we used a gene regulatory network constructed by a text mining approach^9^ and complemented it with TF – gene interactions from the public TRANSFAC database^17^. The construction of the text mining network included an extensive manual curation step, thus highly improving the reliability of the detected relations compared to a completely automated approach. In addition to the text-mining, the network also contains interactions reported in the TRANSFAC database, which based on biological experiments. In *E. coli*, the network was retrieved from RegulonDB^18^, a gold standard in the field. We therefore believe that both the human and the *E. coli* network represent a pertinent choice to provide background knowledge to the methods. Further, the use of other networks (restricted versions of the original human and *E. coli* networks or networks inferred by ARACNE) did not improve or change substantially the results.

In general, the selection of experiments might affect the outcome of the methods. We used experiments from the GEO platform, an established and extensive repository for genomic data sets, to ensure an easy and public access to the data and to allow other researchers to replicate our results. We chose data from different species, different cells of origin and cell lines, from various contributors, data measured on different arrays, and of 13 different KD TFs to ensure that our results are generalizable and depend less on the specific datasets. However, the chosen experiments had to fulfill certain criteria: obviously, they had to contain a KD scenario and we chose to include only experiments with at least three samples per condition. Further, as we wanted to include ISMARA as a method for estimating TF activity, we had to choose experiments whose Affymetrix chips were supported by its web service. All these constraints limited the number of possible data sets. Of course, the use of other experimental data, different underlying networks or additional methods might affect the results. However, since we draw our conclusions from a total of 25 evaluated data sets, amongst which we did not detect a pattern justifying an especially good or bad performance, we argue that our results show not only individual artefacts but are generalizable to the estimation of TF activity in KD studies.

Altogether, the investigated methods for estimating TF activity were not able to robustly detect knocked down TFs neither in human nor in *E. coli* data. We believe that the main reason for this deficiency is the simplistic model of cellular processes used in the more complex methods like ISMARA. We can only speculate which aspects are primarily responsible for the limited performance. All considered methods only used gene expression data whereas other important regulatory processes such as epigenetic mechanisms like DNA methylation, chromatin remodeling, complex promoter structures, and posttranscriptional regulatory processes via microRNAs are disregarded. The inclusion of further data types would probably change the outcome of the methods and might improve results^8^. Also, all models assume linear relationships between TFs and lack a notion of kinetic or temporal effects^36^. Although time series expression data from TF knockdown or TF induction experiments exist^37,38^, the selected methods cannot make use of this type of data. Another possible reason for the failure of the methods might be their inability to model TF self-regulation and feedback loops despite their known importance for gene regulation^39,40^.

## Materials and Methods

### Transcriptome data

We downloaded publicly available transcriptome data for different TF knockdowns in human and *E. coli* cell lines from the GEO repository^11^. We chose three different experiments for TF silencing in human cell lines (GSE45838^12^, GSE17172^13,14^, GSE19114^15^) and one experiment in *E. coli* (GSE1121^16^). They contain data from 8 human knocked down genes (*BCL6*, *FOXM1*, *MYB*, *bHLH-B2*, *FOSL2*, *RUNX1*, *C/EBPβ*, *STAT3* and double knockdown *C/EBPβ & STAT3*) and 5 knocked down genes (*AppY*, *ArcA*, *Fnr*, *OxyR*, *SoxS* and double knockdown *ArcA & Fnr*) from *E. coli*. PCA plots for all data sets are provided in Supplementary Fig. S5 showing the separation of treated and control samples. We mapped the given probe identifiers to HGNC Symbols (human data) or gene symbols from UniGene (*E. coli*). When multiple probes mapped to one gene we computed a t-test comparing case and control group and kept the probe with smallest p-value.

*GSE45838*^12^ contains data from the knock-down of *BCL6* expression in human diffuse Large B-Cell Lymphoma cell lines. This experiment was performed in OCI-Ly7 and Pfeiffer GCB-DLBCL cell lines as triplicates, providing three case and three control samples per cell line. Gene expression was profiled on H-GU133plus2 Affymetrix gene chips. We analyzed the samples in dependence of their cell line origin and treated them as two independent data sets since they were clearly separated in a PCA plot (see Supplementary Fig. S5, panel a).

*GSE17172*^13,14^ consists of samples of Human Burkitt’s lymphoma ST486 cells which were transduced either with non-target control shRNA lentiviral vectors, *FOXM1* shRNA or *MYB* shRNA lentiviral vectors (three samples in each condition). cRNA was hybridized in Affymetrix Human Genome U95 Version 2 Arrays. We used the MAS5^41^ normalized data as provided on GEO.

*GSE19114*^15^ includes 74 samples from knockdown experiments in human glioma cell line SNB19 and glioblastoma multiforme-derived brain tumour initiating cells (BTICs). shRNA-mediated silencing targeted *bHLH-B2*, *FOSL2*, *RUNX1*, *C/EBPβ* and *STAT3*. For SNB19, 10 control samples were available together with 4 samples with *bHLH-B2* knockdown, 4 with *FOSL2* knockdown and 3 samples each for *C/EBPβ*, *STAT3* and the combined *C/EBPβ & STAT3* knockdown. Data was available for *C/EBPβ*, *STAT3*, combined *C/EBPβ & STAT3* knockdown and a control condition for 11 samples in each group in BTICs. RNA was hybridized on Illumina HumanHT-12v3 expression BeadChip. Since the samples were clearly separated in a PCA plot by their cell type (see Supplementary Fig. S5, panel c), we treated data from SNB19 and BTICs independently.

*GSE1121*^16^ contains three samples of six *E. coli* strains with knockouts of transcriptional regulators in the oxygen response (*AppY*, *ArcA*, *Fnr*, *OxyR*, *SoxS* and the double knockout *ArcA & Fnr*) in both aerobic and anaerobic conditions. Additionally, three (aerobic condition) and four (anaerobic condition) wild type samples were available. Gene expression was profiled on Affymetrix *E. coli* Antisense Genome Arrays. We analyzed the data from the two oxygen conditions independently.

### TF – gene network

We provided a human TF – gene network^8,9^ and one from *E. coli*^18^ as input for biRte, RABIT and RACER, which are available as supplementary files. ISMARA can only be used with its own underlying regulatory network model, which is not accessible explicitly.

#### Human

We used a publicly available TF – gene network^9^ based on a text-mining approach complemented with TF – gene interactions from the public TRANSFAC database^17^ (http://www.gene-regulation.com/pub/databases.html, release 7.0). This network includes 2894 interactions between 429 TFs and 1218 genes. The network is provided as supplementary material.

#### E. coli

We downloaded TF – gene interactions from RegulonDB^18^ version 9.0, Release 9.4 and kept those interactions for which at least one entry in the column “Evidence that supports the existence of the regulatory interaction” was mentioned. The network contains 4273 interactions between 206 TFs and 1798 genes and is provided as supplementary material.

### Methods for estimating TF activity

The considered methods for estimating TF activity model genome-wide gene regulation as sets of equations over the activity of transcription factors. All methods assume both the set of TFs and the topology of the regulatory network to be given. By combining this background knowledge with transcriptome data, they try to infer the activity of regulators in a certain experimental condition or disease using mathematical optimization to find parameters minimizing the divergence of predicted and measured gene expression intensities. The methods predominantly produce a ranked list of TFs, sorted by their activity in a given group of samples. Here, we briefly explain the functioning of each method and state our parameter settings if applicable. For a detailed description of each method refer our previously published review^8^ or the according original paper. The investigated methods are publically available as web service, downloadable program or R package.

### BiRte

BiRte^4^ (Bayesian inference of context-specific regulator activities) uses a probabilistic framework to estimate regulatory activities from differential gene expression data and a TF – gene network. The set of active regulators can be seen as hidden state variables which are estimated with help of the Markov Chain Monte Carlo method. Thereby, the posterior probability for each regulator and condition to influence the expression of its target genes is estimated. Simultaneously, a variable selection procedure is applied to achieve sparsity of the model.

BiRte is available as a bioconductor R package. We used R version 3.2.0 with biRte version 1.10.0 and applied the method “birteLimma” to estimate regulatory activities with the options “niter” and “nburnin” set to 10000. As biRte has a randomized component, the resulting TF activities are not exactly the same for different runs. We averaged the final activity scores over 100 iterations of birteLimma.

### ISMARA

ISMARA^5^ (Integrated System for Motif Activity Response Analysis) infers the activity of regulatory motifs (short nucleotide sequences) and thereby indirectly deduces the effects of TFs. The input signal levels, which are computed from gene expression data, are modelled linearly in terms of binding site predictions and unknown motif activities. ISMARA employs a Bayesian procedure with a Gaussian likelihood model and a Gaussian prior distribution for inferred motif activity profiles to avoid overfitting.

In contrast to all other investigated methods, TF – gene relationships are not provided by the user. ISMARA can only be run with its proprietary underlying regulatory network model. Further, only raw data provided as CEL, FASTQ, BED, BAM or SAM files can be uploaded. Therefore, the results from ISMARA are only partly comparable to the results from other methods here.

ISMARA is available via a web service (https://ismara.unibas.ch/fcgi/mara). We uploaded raw CEL files and grouped the samples according to their origin or treatment to compare the average regulatory activity between different conditions. Since Illumina chips in general and the Affymetrix *E. coli* Antisense Genome Array in particular are not supported, we could only run ISMARA on the data sets with BCL6 (GSE45838) and FOXM1/MYB (GSE17172) knockdown.

### RABIT

RABIT^6^ (Regression Analysis with Background Integration) applies a linear regression model to estimate TF activities. First, RABIT tests in each sample whether the target genes of each TF are differentially expressed. A score indicating regulatory activity is defined by the t-value (regression coefficient divided by standard error). To find a subset of TFs among those screened before, a stepwise forward selection is applied optimizing the model error. Lastly, TFs with insignificant cross-sample correlation are removed from the results.

The authors of RABIT published a C++ implementation accessible under http://rabit.dfci.harvard.edu which we adopted with the FDR option set to 1. We used the difference of expression values between case and control group as input and ordered the activity of TFs by t-value as proposed in the RABIT paper.

### RACER

RACER^7^ (Regression Analysis of Combined Expression Regulation) consists of a two-stage linear regression. Optimization is applied twice to reduce model complexity, where the method first infers sample-specific TF activities and uses these, in a second step, to compute general TF – gene interactions. Sparsity of the solution is obtained through elastic-net regularized generalized linear models. A supplementary feature selection procedure comparing the full model to a restricted model leaving one TF out provides the most predominant regulators.

We used the available R scripts from http://www.cs.utoronto.ca/~yueli/racer.html to run RACER and set miRNA expression data, copy number variation and methylation scores, which have to be provided, to zero. The obligatory miRNA – gene network was artificially created where all dummy miRNAs and genes were connected. We computed separate models for case and control group and extracted the resulting sample-specific regulatory activities. TFs were ranked by their activity difference between the two groups.

### Ranking

For all ranking assignments, we appointed TFs that compared equal the same rank. Subsequently, a gap was left in the ranking numbers which size was equal to the number of items that compared equal minus 1.

#### Differential expression ranking

We calculated the differential expression between case and control group for all genes in all data sets via a two-sided t-test. We ranked the genes according to the p-value of the t-test (smallest p-value corresponds to rank 1). We did not apply any multiple test correction, since we were not interested in the precise p-value but only the order of p-values to assign ranks.

#### TF activity score ranking

In each data set, we compared the results of each method by ranking the absolute values of the computed TF activity scores (highest absolute activity value corresponds to rank 1). Activities equal to zero were not considered. Therefore, the total number of ranked TFs is different in each method and data set. We predominantly assessed the rank of the TF that was knocked down in particular. Additionally to the KD TF, we evaluated the ranks (if existing) of

- directly connected TFs in the network
- aliases provided in the GeneCards^23^ database version 4.8.0 Build 5 (available under www.genecards.org) for human TFs respectively synonyms from the EcoCyc^21^ database (ecocyc.org) for *E. coli*.
- TFs directly connected in a pathway from SignaLink^20^ 2.0 (signalink.org) for human TFs respectively from the EcoCyc^21^ database (ecocyc.org) for *E. coli*.
- TFs directly interacting with the KD TF given in TcoF-DB^22^ version 2.2.2 available under http://tools.sschmeier.com/tcof/home for human.

We call the union of these TFs related TFs. An overview is available in Supplementary Table S2. The table shows all TFs that were found, irrespective whether they appear in our regulatory networks or not. For each method and data set individually, we evaluated whether the resulting ranks of all related TFs are significantly smaller than the average rank. We applied a one-sided one-sample t-test to compare the mean rank against the average rank (total number of ranked TFs divided by 2) and considered p-values < 0.05 as significant. Since the total number of t-test is still quite small (54) and nearly all p-values were above the significance level anyway, we did not apply multiple testing correction.

#### Overlap

We computed the number of overlapping TFs in the top 10 and top 100 lists comparing all applied methods within one data set. The resulting Venn diagrams are shown in Supplementary Fig. S3.

#### Small networks

To evaluate the influence of the human gene regulatory network, we reduced the network to the close neighborhood of the knocked down TF with a distance of 2, thus giving smaller gene regulatory networks for each knocked down TF.

#### ARACNE

We applied ARACNE^19^ (Algorithm for the Reconstruction of Accurate Cellular Networks) to reconstruct a gene regulatory network using the *FOXM1* (human) and *AppY* (*E. coli*) KD transcriptome data. We used the implementation of the “minet” bioconductor package^42^ in R (version 3.38) and built the mutual information matrix with Spearman’s correlation. The threshold for removing an edge in the aracne function was set to 0.1. We used the resulting gene regulatory networks as input to biRte^4^, RABIT^6^ and RACER^7^ and ranked the estimated TF activity scores as described above.

## Supplementary information

Supplementary Information

Dataset 1

Dataset 2

## Data Availability

The data sets analyzed during the current study are available in the GEO repository under the following accession codes: GSE45838, GSE17172, GSE19114 and GSE1121. Human TF – gene interactions were obtained from text mining based on the paper by Thomas *et al*.^9^ (available via the FastForward DNA database under http://fastforward.sys-bio.net) and complemented with interactions from the TRANSFAC database (release 7.0, http://www.gene-regulation.com/pub/databases.html). The network is provided as supplementary material in file “human_tf_gene_network.txt”. The TF – gene network for *E. coli* was downloaded from RegulonDB^18^ version 9.0, Release 9.4 (http://regulondb.ccg.unam.mx/menu/download/datasets/index.jsp). The network is provided as supplementary material in file “ecoli_tf_gene_network.txt”.
