# Supplementary material for: Estimation of Transcription Factor Activity in Knockdown Studies
Source: Sci Rep. 2019 Jul 3;9:9593. doi: 10.1038/s41598-019-46053-7 (PMC6610105; doi:10.1038/s41598-019-46053-7)
Supplement: Supplementary file 1 — Supplementary Information [file 41598_2019_46053_MOESM1_ESM.pdf]

Estimation of Transcription Factor Activity in Knockdown Studies

Saskia Trescher[1]*, Ulf Leser[1]

[1]Knowledge Management in Bioinformatics, Computer Science Department, Humboldt-Universität zu Berlin, Unter den Linden 6, 10099 Berlin, Germany

Email Addresses:
saskia.trescher@informatik.hu-berlin.de
leser@informatik.hu-berlin.de

*Corresponding Author

Supplementary Information

Supplementary Figures

Ranks of differential expression

Supplementary Figure S1: Ranks for differential expression of KD TFs and total number of ranked TFs per data set. Differential expression ranks of KD TFs in the top 5% of all ranked TFs are marked in dark orange, ranks in the top 5-10% in orange and ranks in the top 10-20% in light orange. Two ranks in one table cell refer to a combined KD of two TFs and are given in the order of the TFs at the beginning of the table row.

Orga-nism	Experi-ment	TF Knockdown	Cell line/ condition	rank		total
Hu-man	GSE45838	***BCL6***	OCI–Ly7	9		371
			Pfeiffer	5		371
	GSE17172	***FOXM1***	ST486	1		331
		MYB	ST486	1		331
	GSE19114	***bHLH-B2***	SNB19	174		368
		FOSL2	SNB19	108		368
		RUNX1	SNB19	72		368
		C/EBPβ	SNB19	7		368
			BTICs	330		368
		STAT3	SNB19	1		368
			BTICs	1		368
		C/EBPβ & STAT3	SNB19	6/	4	368
			BTICs	117/	1	368
E. coli	GSE1121	***AppY***	aerobic	8		150
			anaerobic	4		150
		ArcA	aerobic	13		149
			anaerobic	7		150
		ArcA & Fnr	aerobic	11/	4	151
			anaerobic	13/	8	151
		Fnr	aerobic	3		151
			anaerobic	3		150
		OxyR	aerobic	1		149
			anaerobic	1		150
		SoxS	aerobic	17		150
			anaerobic	1		150

Ranks of related TFs

Supplementary Figure S2: Ranks of KD TFs (bold) and related TFs, total number of ranked TFs per method and p-value indicating significance of test whether the mean of the ranks of all related TFs is smaller than the average rank (total number of ranks divided by 2). Significant p-values are marked in yellow. Ranks of TFs in the top 5% of all ranked TFs are marked in dark green, ranks in the top 5-10% in green and ranks in the top 10-20% in light green. When a TF was not ranked, "-" is shown. Two ranks in one table cell refer to a combined KD of two TFs and are given in the order of the TFs at the beginning of the table row.

Human

Experiment GSE45838: knockdown of BCL6

Cell line	OCI–Ly7				Pfeiffer			
TF \ Method	biRte	ISMARA	RABIT	RACER	biRte	ISMARA	RABIT	RACER
BCL6	**266**	-	-	-	**163**	-	-	**68**
TP53	397	138	-	-	-	235	-	-
FOXO4	49	374	-	33	1	97	-	-
SPI1	116	146	-	-	-	3	-	-
IRF4	36	-	17	-	117	-	-	-
JUN	-	321	39	83	386	116	-	-
JUNB	147	-	-	-	376	-	-	-
JUND	-	167	-	-	7	253	-	-
TWIST1	332	-	-	-	78	-	29	-
ZBTB16	377	392	-	11	179	485	-	42
ZBTB7A	282	-	-	-	397	-	-	-
ZBTB7B	353	-	-	10	262	-	-	-
total	404	500	58	88	405	500	53	143
p-value	0.770	0.512	0.471	0.368	0.453	0.181	-	0.212

Experiment GSE17172: knockdown of FOXM1

Cell line	ST486			
TF \ Method	biRte	ISMARA	RABIT	RACER
FOXM1	**9**	-	-	-
ESR1	387	218	22	-
TP53	259	310	-	-
SMAD3	113	62	-	-
SP1	339	555	-	-
ZBTB3	-	558	-	-
total	398	602	63	4
p-value	0.617	0.649	-	-

Experiment GSE19114: knockdown of bHLH-B2, FOSL2, RUNX1, C/EBPβ, STAT3 and C/EBPβ & STAT3

Cell line	SNB19		
TF \ Method	biRte	RABIT	RACER
bHLH-B2	**186**	-	-
ARNT	202	21	-
ID1	237	-	-

TP53	54	37	-			
TP63	326	-	-			
TP73	146	-	-			
ARNT.HIF1A	263	-	-			
BRCA1	6	6	-			
ENO1	223	-	-			
HIVEP1	155	-	-			
TCF3	203	-	-			
total	402	42	0			
p-value	0.136	0.513	-			
Method / TF	biRte	RABIT	RACER			
FOSL2	355	-	-			
BRCA1	237	-	-			
FOSL1	276	54	-			
ATF2	321	-	-			
ATF3	384	11	-			
JUN	365	-	-			
JUNB	229	-	-			
JUND	261	-	-			
total	404	54	0			
p-value	0.999	0.58	-			
Method / TF	biRte	RABIT	RACER			
RUNX1	8	37	-			
FOXP3	213	-	-			
NFE2	271	6	-			
RUNX3	56	-	-			
CBFB	371	-	-			
CEBPB	6	17	-			
ELF1	251	-	-			
ELF2	9	1	-			
ELF4	257	-	-			
FOS	2	11	-			
MYOD1	346	-	-			
PAX5	255	-	-			
VDR	233	-	-			
total	401	49	0			
p-value	0.261	0.09	-			
Cell line	BTICs			SNB19		
Method / TF	biRte	RABIT	RACER	biRte	RABIT	RACER
CEBPB	328	-	-	-	-	-
RUNX2	-	-	-	290	-	-

JUN	385	-	-	394	-	-
TP63	56	-	-	-	-	-
BRCA1	262	-	-	166	-	-
AR	129	-	-	397	44	-
ATF3	238	6	-	268	-	-
ATF4	346	36	-	160	-	-
CEBPA	322	-	-	244	-	-
CEBPD	320	-	-	311	-	-
CREB1	269	-	-	-	-	-
EGR1	323	-	-	336	-	-
ESR1	370	44	-	371	-	-
FOXO1	90	-	-	365	-	-
HMGA1	200	-	-	347	-	-
HSF1	87	-	-	26	-	-
KLF5	291	-	-	28	38	-
NFKB1	118	-	-	-	-	-
NR3C1	-	-	-	143	41	-
PPARG	168	-	-	174	-	-
RARB	314	-	-	116	-	-
RELA	-	-	-	227	-	-
RUNX1	281	-	-	398	-	-
SMAD3	276	-	-	281	-	-
SMAD4	335	-	-	366	-	-
SMARCA2	306	-	-	291	-	-
SPI1	-	-	-	167	-	-
Max rank	397	61	14	404	49	0
p-value	0.989	0.444	-	0.982	0.995	-

Cell line	BTICs			SNB19		
Method / TF	biRte	RABIT	RACER	biRte	RABIT	RACER
STAT3	209	-	-	4	29	-
HOXA1	1	58	-	5	21	-
CEBPD	223	55	-	25	-	-
FOS	88	-	-	396	-	-
IRF1	213	-	-	-	-	-
MUC1	298	-	-	75	-	-
SREBF1	309	-	-	7	38	-
TP53	-	-	-	167	55	-
TP63	202	-	-	289	-	-
ESR1	-	-	-	326	-	-
ETS1	393	-	-	238	-	-
FOXA1	131	-	-	383	-	-
INSM1	343	-	-	289	-	-
NR2F1	346	-	-	377	-	-

STAT1	-	-	-	81	-	-
TCF3	157	-	-	35	-	-
TEAD1	321	51	-	176	-	-
VDR	170	-	-	403	-	-
AR	369	-	-	-	-	-
ATF3	123	56	-	257	-	-
GTF2I	54	-	-	72	-	-
HES1	332	-	-	363	-	-
HIVEP1	156	-	-	344	-	-
KLF15	178	-	-	288	-	-
MYOD1	162	-	-	400	-	-
NCOA1	383	-	-	225	-	-
NFKB1	356	-	-	323	18	-
NR4A1	82	17	-	351	-	-
PPARD	128	-	-	123	-	-
RELA	395	16	-	-	-	-
STAT6	103	-	-	102	-	-
TWIST1	11	-	-	2	23	-
ZNF281	184	-	-	31	33	-
total	405	60	14	403	59	0
p-value	0.700	0.902	-	0.562	0.617	-

Cell line	BTICs			SNB19		
Method / TF	biRte	RABIT	RACER	biRte	RABIT	RACER
STAT3	**188**	-	-	31	-	-
CEBPB	**402**	-	-	-	-	-
HOXA1	3	-	-	70	-	-
CEBPD	356	-	-	290	-	-
FOS	150	-	-	359	-	-
IRF1	71	47	-	-	-	-
MUC1	122	-	-	267	-	-
SREBF1	357	-	-	396	-	-
TP53	-	-	-	373	-	-
TP63	332	-	-	185	-	-
ESR1	232	-	-	-	-	-
ETS1	380	-	-	-	-	-
FOXA1	355	-	-	351	-	-
INSM1	320	-	-	82	-	-
NR2F1	370	-	-	52	-	-
STAT1	347	-	-	-	-	-
TCF3	336	-	-	138	-	-
TEAD1	322	-	-	273	-	-
VDR	40	60	-	312	-	-
AR	191	-	-	149	-	-

ATF3	353	8	-	-	-	-
GTF2I	57	-	-	369	-	-
HES1	186	-	-	216	-	-
HIVEP1	95	-	-	78	-	-
JUN	-	-	-	393	-	-
KLF15	304	-	-	183	-	-
MYOD1	70	-	-	204	-	-
NCOA1	133	-	-	334	-	-
NFKB1	45	-	-	253	-	-
NR3C1	403	42	-	320	-	-
NR4A1	124	16	-	200	-	-
PPARD	160	-	-	177	-	-
RELA	272	65	-	-	-	-
STAT6	299	-	-	197	-	-
TWIST1	8	2	-	3	3	-
ZNF281	178	-	-	311	-	-
RUNX2	309	-	-	368	-	-
BRCA1	17	34	-	300	-	-
ATF4	262	21	-	281	-	-
CREB1	391	-	-	387	-	-
EGR1	204	-	-	-	-	-
FOXO1	-	-	-	371	-	-
HMGA1	243	-	-	239	-	-
HSF1	288	-	-	54	-	-
KLF5	311	-	-	256	-	-
PPARG	406	-	-	106	50	-
RARB	146	-	-	102	-	-
RUNX1	201	-	-	-	-	-
SMAD3	22	41	-	-	28	-
SMAD4	20	37	-	-	-	-
SMARCA2	164	-	-	111	-	-
SPI1	69	-	-	394	-	-
SRF	405	-	-	373	-	-
total	410	71	14	400	51	0
p-value	0.797	0.40	-	0.972	0.539	-

E. coli

Experiment GSE1121: knockdown of AppY, ArcA, Arca & Fnr, Fnr, OxyR and Soxs

Condition	aerobic			anaerobic		
Method / TF	**biRte**	**RABIT**	**RACER**	**biRte**	**RABIT**	**RACER**
AppY	**119**	**-**	**73**	**15**	**1**	**71**
DpiA	148	-	-	24	-	-
H-NS	154	-	137	169	-	-

ArcA	14	21	43	1	24	64
total	199	48	152	198	43	121
p-value	0.603	-	0.604	0.159	0.289	0.852

Condition	aerobic			anaerobic		
Method / TF	**biRte**	**RABIT**	**RACER**	**biRte**	**RABIT**	**RACER**
ArcA	**198**	**-**	**70**	**1**	**2**	**135**
Fnr	197	-	-	195	-	138
total	198	32	142	199	42	147
p-value	0.998	-	-	0.495	-	0.992

Condition	aerobic			anaerobic		
Method / TF	**biRte**	**RABIT**	**RACER**	**biRte**	**RABIT**	**RACER**
ArcA	**6**	**5**	**108**	**1**	**1**	**34**
Fnr	**7**	**6**	**-**	**148**	**-**	**104**
Fur	1	1	105	12	14	41
IHF	184	12	71	196	20	105
SoxS	14	24	103	198	-	101
total	199	29	133	198	45	115
p-value	0.091	0.144	0.98	0.597	0.097	0.853

Condition	aerobic			anaerobic		
Method / TF	**biRte**	**RABIT**	**RACER**	**biRte**	**RABIT**	**RACER**
Fnr	**9**	**10**	**-**	**192**	**43**	**127**
ArcA	7	2	99	1	6	37
Fur	1	1	118	46	45	51
IHF	186	18	59	196	31	111
SoxS	58	-	110	197	-	79
total	199	33	137	199	55	143
p-value	0.124	0.057	0.939	0.719	0.648	0.696

Condition	aerobic			anaerobic		
Method / TF	**biRte**	**RABIT**	**RACER**	**biRte**	**RABIT**	**RACER**
OxyR	**7**	**28**	**83**	**6**	**10**	**94**
CRP	179	-	107	5	-	-
total	197	34	135	199	35	121
p-value	0.48	-	0.869	0.002	-	-

Condition	aerobic			anaerobic		
Method / TF	**biRte**	**RABIT**	**RACER**	**biRte**	**RABIT**	**RACER**
SoxS	**1**	**10**	**95**	**14**	**-**	**92**
SoxR	11	-	-	1	-	-
AcrR	139	-	47	163	-	57
Fnr	190	15	-	53	5	101

Fur	1	-	52	182	12	37
total	199	40	146	199	45	119
p-value	0.241	0.102	0.32	0.337	0.078	0.763

Overlap of top 100 regulators

Supplementary Figure S3: Venn diagrams with number of overlapping TFs in the top 100 by estimating TF activity with different methods. For RABIT and RACER, the total number of ranked TFs was below 100 in some cases (see Fig. 3).

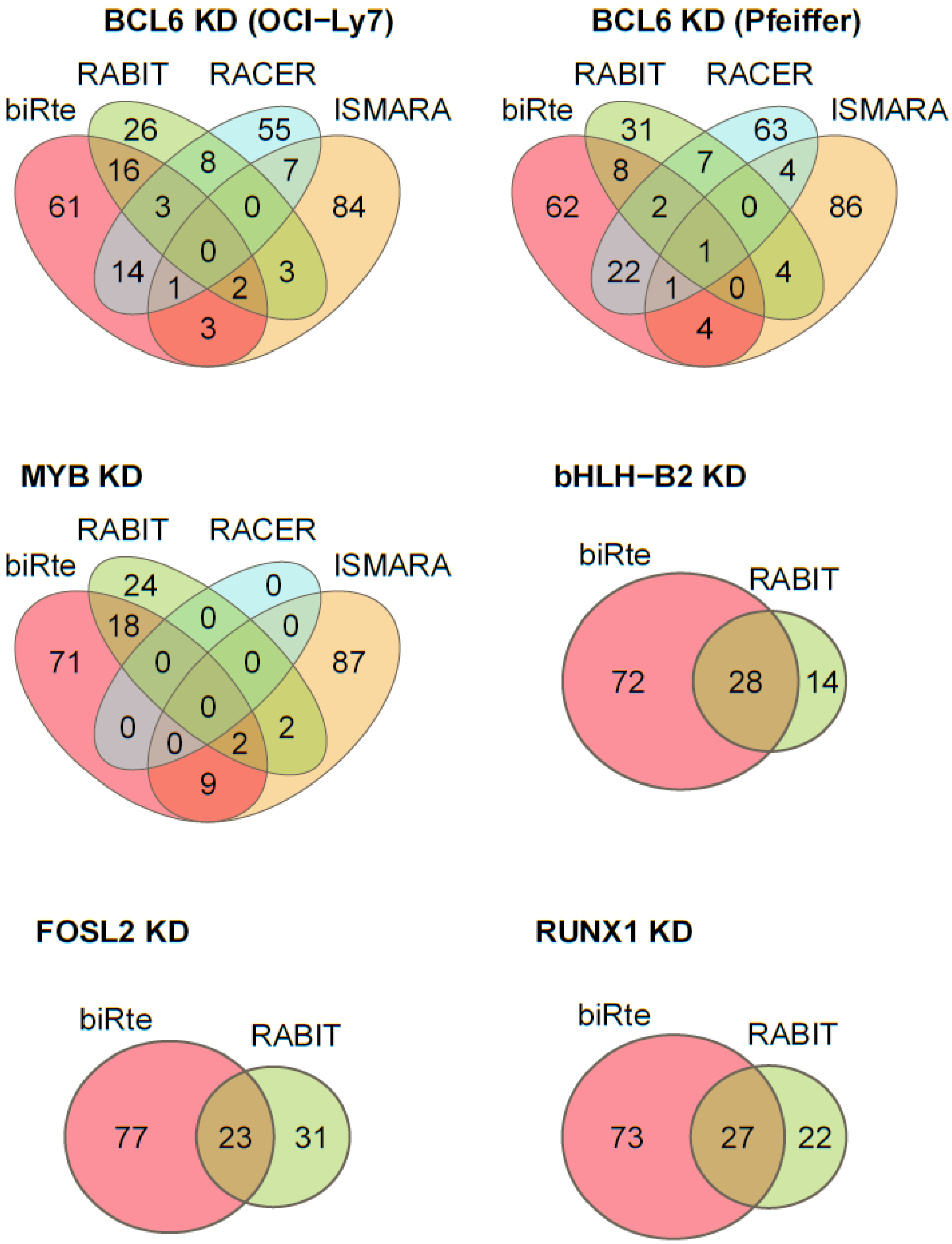

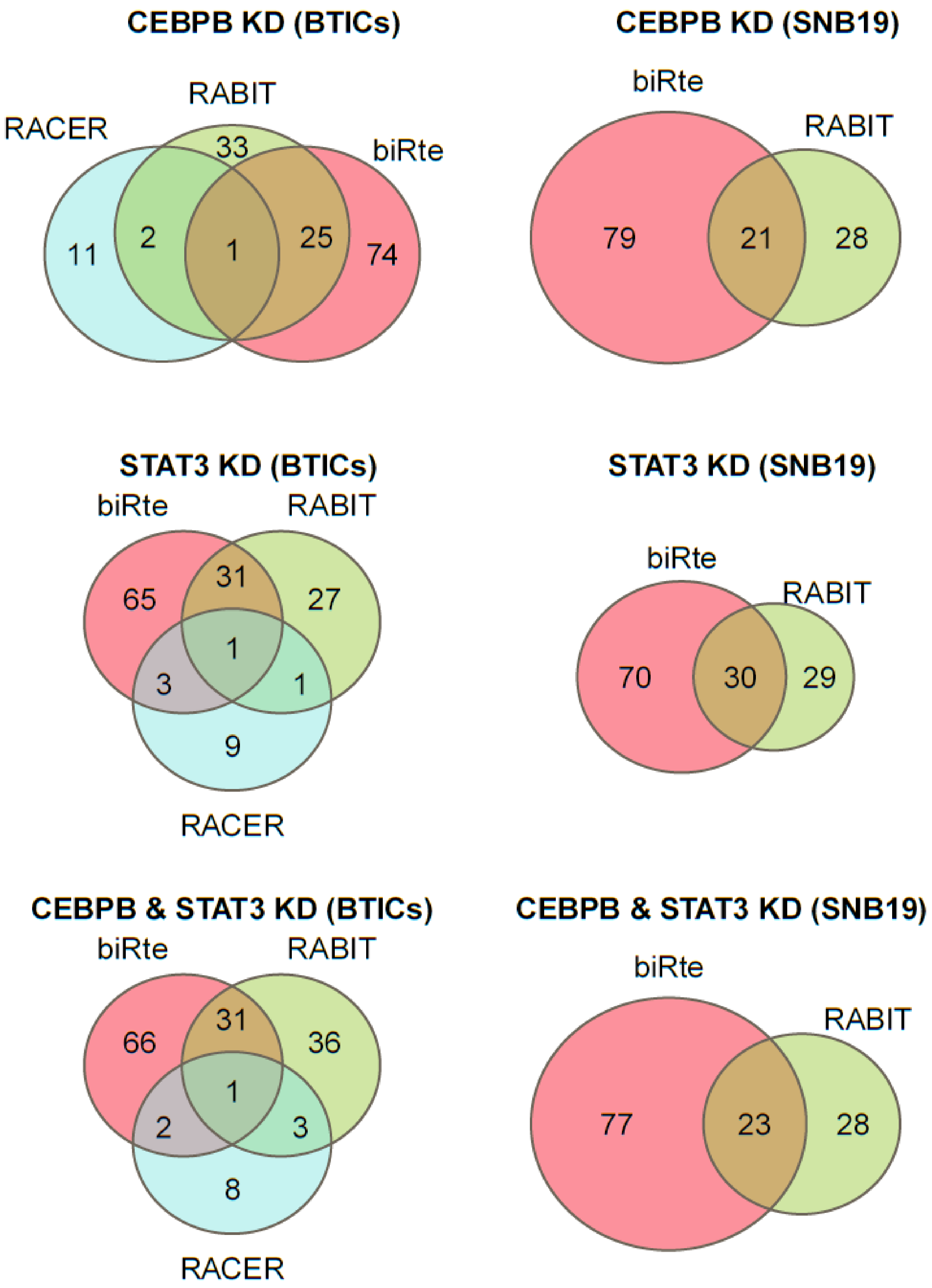
CEBPB KD (BTICs)
RACER
RABIT
biRte
33
11
2
1
25
74
CEBPB KD (SNB19)
biRte
RABIT
79
21
28
STAT3 KD (BTICs)
biRte
RABIT
65
31
27
1
3
1
9
RACER
STAT3 KD (SNB19)
biRte
RABIT
70
30
29
CEBPB & STAT3 KD (BTICs)
biRte
RABIT
66
31
36
1
2
3
8
RACER
CEBPB & STAT3 KD (SNB19)
biRte
RABIT
77
23
28

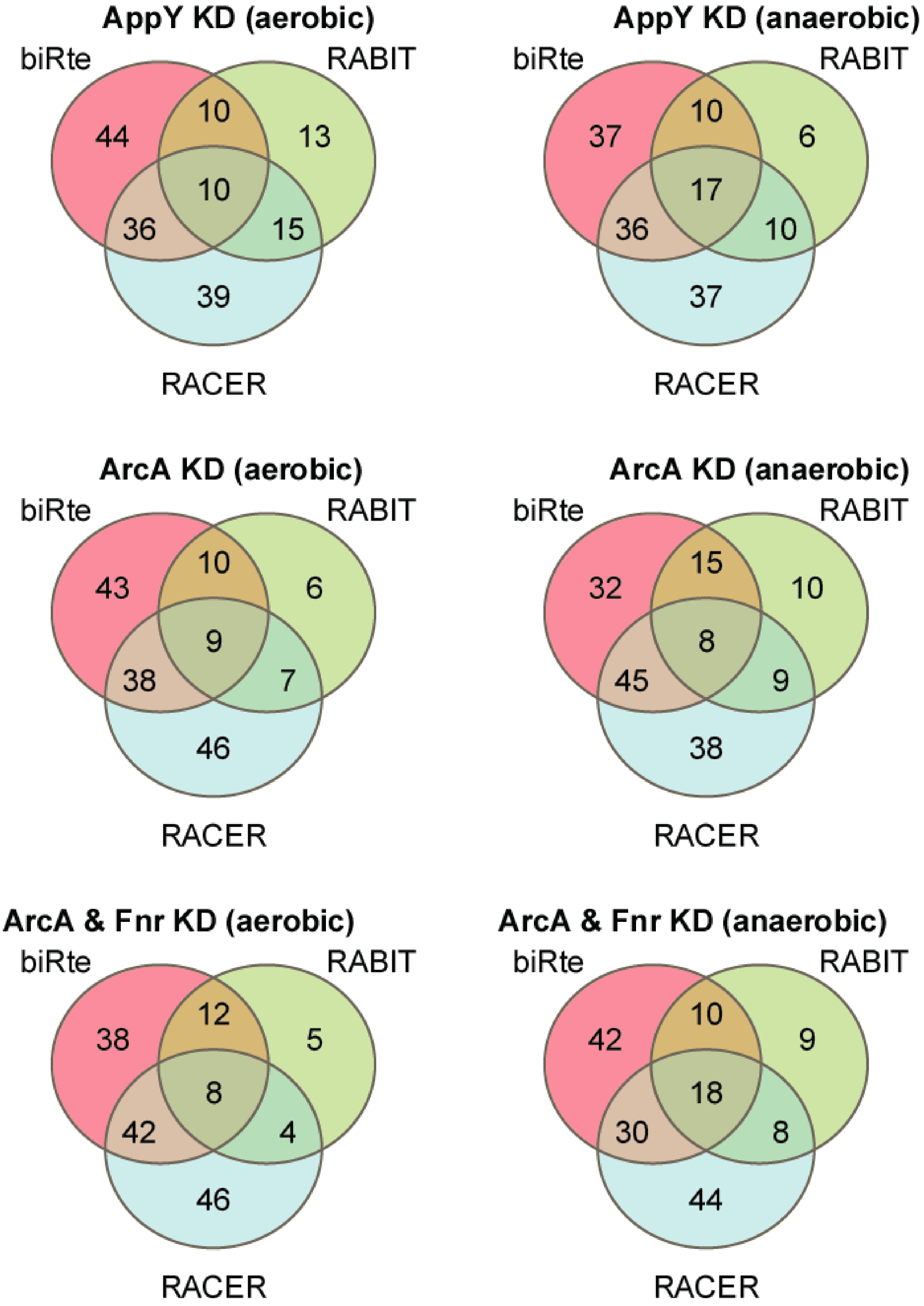
AppY KD (aerobic)
biRte
RABIT
44
10
13
10
36
15
39
RACER
AppY KD (anaerobic)
biRte
RABIT
37
10
6
17
36
10
37
RACER
ArcA KD (aerobic)
biRte
RABIT
43
10
6
9
38
7
46
RACER
ArcA KD (anaerobic)
biRte
RABIT
32
15
10
8
45
9
38
RACER
ArcA & Fnr KD (aerobic)
biRte
RABIT
38
12
5
8
42
4
46
RACER
ArcA & Fnr KD (anaerobic)
biRte
RABIT
42
10
9
18
30
8
44
RACER

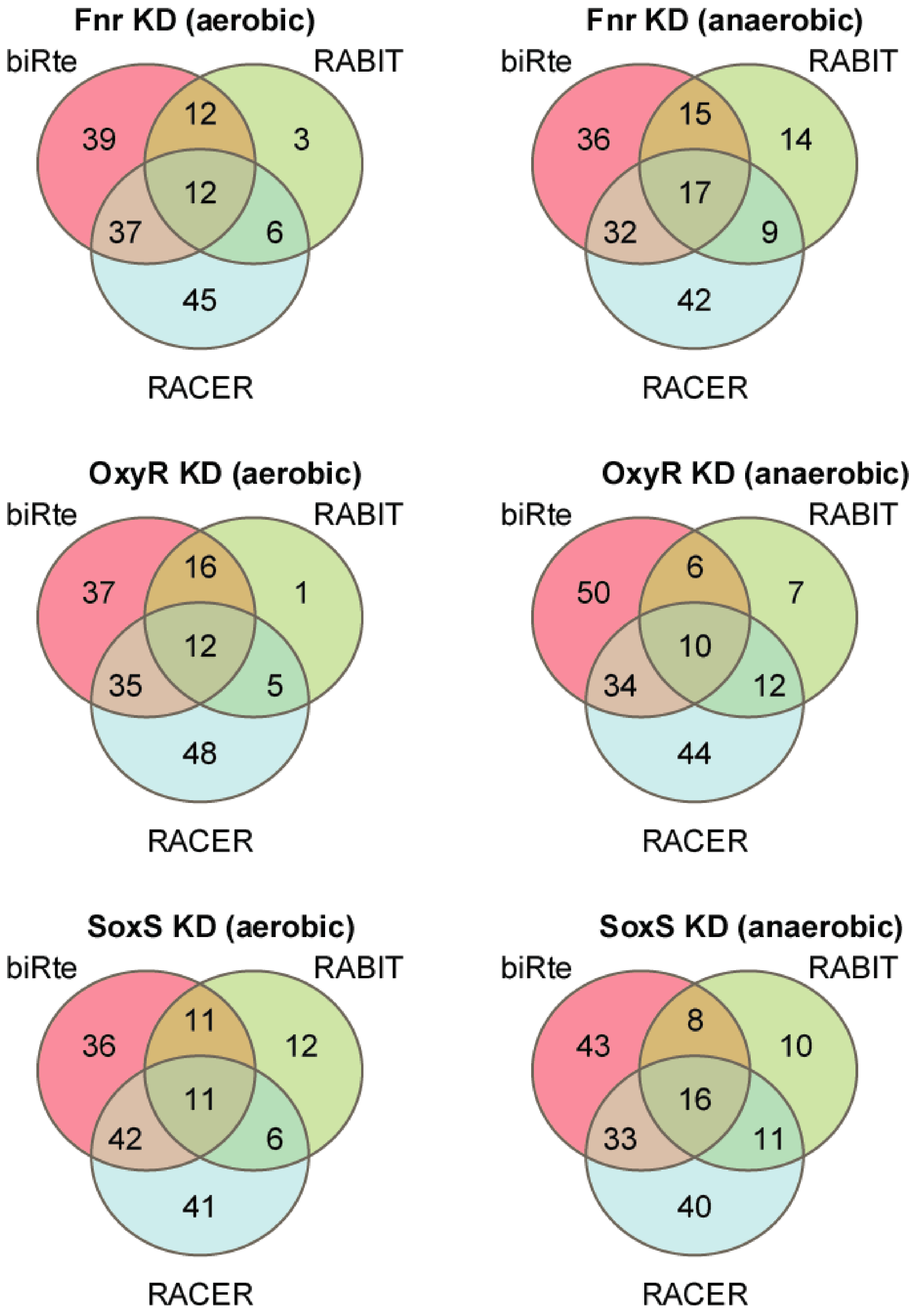
Fnr KD (aerobic)
biRte
RABIT
39
12
3
12
37
6
45
RACER
Fnr KD (anaerobic)
biRte
RABIT
36
15
14
17
32
9
42
RACER
OxyR KD (aerobic)
biRte
RABIT
37
16
1
12
35
5
48
RACER
OxyR KD (anaerobic)
biRte
RABIT
50
6
7
10
34
12
44
RACER
SoxS KD (aerobic)
biRte
RABIT
36
11
12
11
42
6
41
RACER
SoxS KD (anaerobic)
biRte
RABIT
43
8
10
16
33
11
40
RACER

Ranks of TF activity with ARACNE network

Supplementary Figure S4: Ranks of KD TFs (bold) and total number of ranked TFs per method using a network inferred by ARACNE as input. Ranks of TFs in the top 5% of all ranked TFs are marked in green and ranks in the top 5-10% in light green. When a TF was not ranked, "-" is shown.

Orga-nism	Experiment	TF Knockdown	Cell line/ condition	biRte		RABIT		RACER	
				rank	total	rank	total	rank	total
Human	GSE17172	***FOXM1***	ST486	9	248	-	70	-	178
E. coli	GSE1121	***AppY***	aerobic	15	145	-	19	21	103

PCA

Supplementary Figure S5: PCA plots (showing first and second component) for all considered data sets. a) GSE45838 (BCL6 *knockdown), b) GSE17172* (FOXM1 *and* MYB *knockdown), c) GSE19114* (C/EBPβ, STAT3, bHLH-B2, FOSL2 *and* RUNX1 *knockdown), d) GSE1121* (ArcA, AppY, Fnr, OxyR *and* SoxS *knockout)*

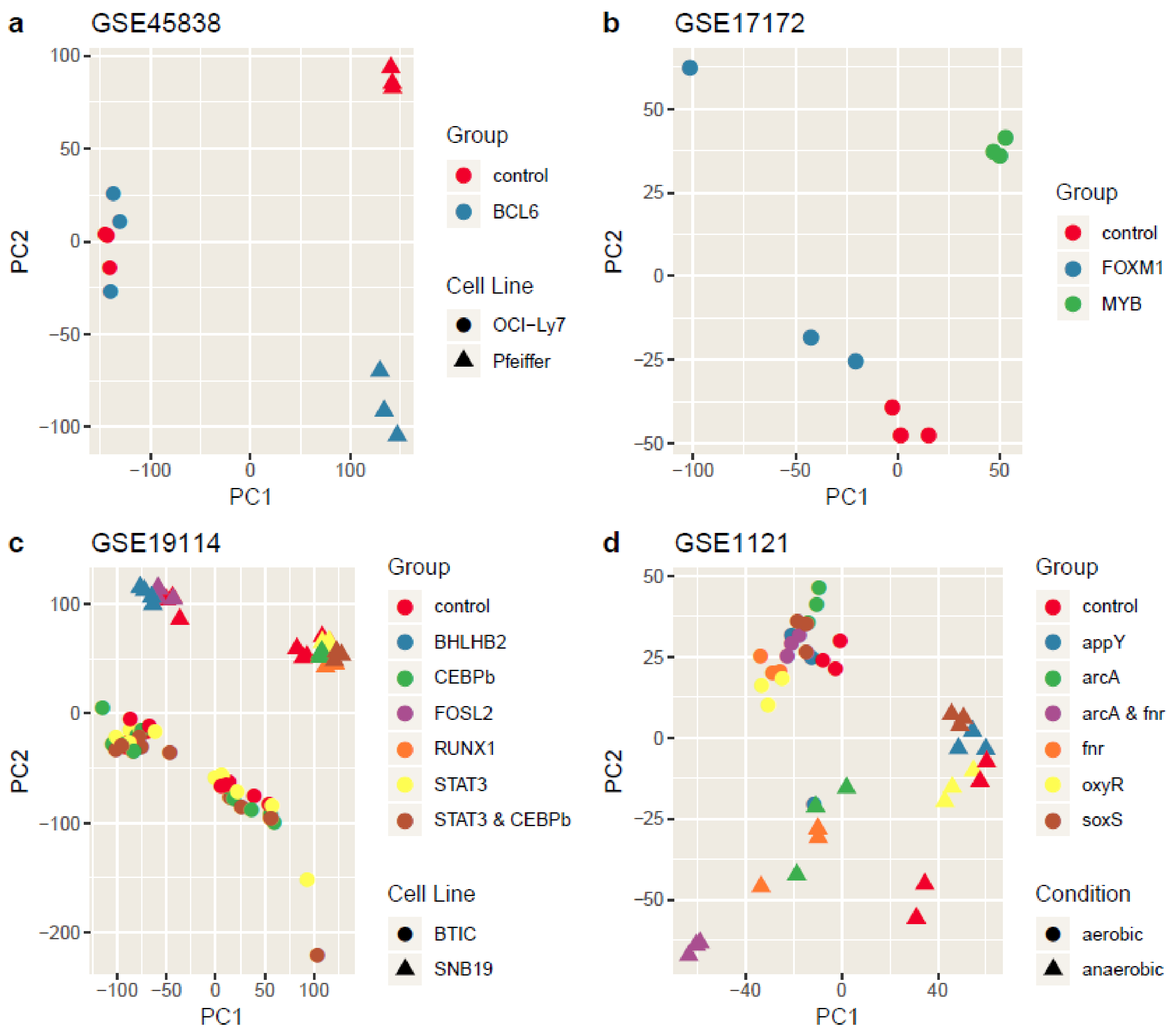

Supplementary Files

TF – Gene networks

The networks are provided as adjacency lists of the connected nodes of the TF – gene network. A list includes three columns (“TF”, “gene”,“edge”) where each row indicates an association with the value of “edge” between a TF and a gene. Complexes of TFs are indicated with a separating “.” between their components.

Human: provided in file “suppl_human_tf_gene_network.csv”

E. coli: provided in file “suppl_ecoli_tf_gene_network.csv”

Supplementary Tables

Significance of differential expression for KD TFs

Supplementary Table S1: p-values for differential expression for KD TFs.

Organism	Experiment	**TF Knockdown**	Cell line/ condition	p-value
Human	GSE45838	***BCL6***	OCI–Ly7	0.001368
			Pfeiffer	0.000097
	GSE17172	***FOXM1***	ST486	0.000360
		MYB	ST486	0.000072
	GSE19114	***bHLH-B2***	SNB19	0.012660
		FOSL2	SNB19	0.009246
		RUNX1	SNB19	0.001226
		C/EBPβ	SNB19	3.94e-07
			BTICs	0.957500
		STAT3	SNB19	1.88e-08
			BTICs	3.42e-14
		C/EBPβ & STAT3	SNB19	3.84e-07 & 4.91e-08
			BTICs	0.05750 & 1.16e-12
E. coli	GSE1121	***AppY***	aerobic	0.027750
			anaerobic	0.010610
		ArcA	aerobic	0.014550
			anaerobic	0.002621
		ArcA & Fnr	aerobic	0.012500 & 0.002288
			anaerobic	0.002174 & 0.001488
		Fnr	aerobic	0.001773
			anaerobic	0.001363
		OxyR	aerobic	0.000020
			anaerobic	7.69e-06
		SoxS	aerobic	0.032830
			anaerobic	0.000152

Related TFs

Supplementary Table S2: TFs related to the knocked down TF.

Human				
**Knockdown TF**	**TFs directly connected in regulatory network**	**Aliases (GeneCards)**	**TFs connected via pathway (SignaLink 2.0)**	**Interactions with other TFs (TcoF DB)**
BCL6	*BCL2L1, CCND2, FCER2, TP53, FOXO4, SPI1*	*ZBTB27, ZNF51, BCL5, LAZ3, BCL6A*	-	*BCL11A, BCL6B, CREBBP, IRF4, JUN, JUNB, JUND, MTA3, NCOR1, NCOR2, PATZ1, SPI1, TP53, TWIST1, ZBTB16, ZBTB7A, ZBTB7B*
FOXM1	*ESR1, TP53, VEGFA, MYC*	*FKHL16, HFH11, MPP2, MPHOSPH2, TRIDENT, FOXM1B, HNF-3, INS-1, MPP-2, PIG29, WIN*	-	*SMAD3, SP1, ZBTB3*
MYB	*ETS1, HOXA9, IRF1, JUN, JUND, ADA, CDK1, COL1A2, GATA3, GSTP1, IGFBP5, KIT, MYC, NR3C1, PAX5, PAX6, PRTN3, SIM2, SLC34A2, SNAI2, SP3, SPP1, SRSF2*	*Cmyb, EFG*	*CCNA1, CCNB1, NR3C1*	*CEBPE, CREBBP, HLF, MAF, NCOR1, SKI, SMARCA2, SP100*
BHLHB2	*ARNT, BHLHE41, ID1, MLH1, PER2, HIF1A, TP53, TP63, TP73, ARNT.HIF1A, ARNTL.CLOCK*	*STRA13, SHARP2, DEC1, BHLHE40, HLHB2*	*APC2, BRCA1, PIM1*	*ENO1, HIVEP1, MYOD1, NOC4L, SOX15, TCF3, ZHX1*
FOSL2	*BRCA1, FOSL1*	*FRA2*	-	*ATF2, ATF3, ATF7, CREB5, DDIT3, JUN, JUNB, JUND, MAFB*
RUNX1	*BCL2, CHI3L1, CLC, CSF1R, CSF2, FOXP3, GATA2, GPR132, GRAP2, IL19, IMPDH2, LAT, LGALS3, NCAM1, NFE2, PLAU, PRKCB, RUNX3*	*CBFA2, AML1, PEBP2aB, AMLCR1, EVI-1*	*ADRA1A, AGTR1, AHI1, ANXA1, ARHGAP39, B4E345, CDK6, CREBBP, EPHA3, GFRA1, GNAQ, IL21, ITGA1, LCP2, MAP3K8, NEK7, PDE3B, PPM1A, PPP2R2A, PRKCA, PRKCB, PRKCW,*	*CBFB, CEBPB, DNMT1, ELF1, ELF2, ELF4, FOS, FOXP3, JUN, KAT6A, MYOD1, NCOR1, PAX5, SPI1, VDR*

			PTPRK, SEC31B, SH3BP5, SPAG16, SYK, TLE1, TRIB1, WDR37, WDR70	
CEBPB	*ABCB1, BCL2A1, BCL2L1, CCL3, CCL4, CCL5, CCR5, CDKN1A, CYP19A1, CYP27B1, DDIT4, DUSP1, F7, FGFBP1, FGFR2, GFER, GLS2, HP, HSD17B8, HSPH1, IL1B, IL1RN, IL5, IL6, INSR, IRF9, LCN2, LDLR, MEFV, MMP1, PLAC1, PRLR, RUNX2, SAA1, SAA2, SLC19A1, SOX6, SPINK1, TNF, TNFAIP6, TNFRSF10, TOP1, TRAF3IP2,YWHAE, CFTR, GPX4, JUN, MMP2, TP63*	*TCF5, IL6DBP, NF-IL6, TCF-5, LAP, LIP*	*APC2, ARMC6, AURKA1, B7Z708, BRCA1, CCNK, CD7, CDC42BPA, CDC7, CLTC, CSTF1, CXCR4, DUSP1, IFT122, ITGB7, NEK3, NEK6, PARD6A, PCNA, PCSK6, PDIK1L, PIM1, POC1A, TRIB1, TRIB3, UBE2F*	*AR, ATF3, ATF4, BATF, BATF3, CEBPA, CEBPD, CEBPG, CREB1, CREBBP, DDIT3, EGR1, ESR1, FOXO1, HMGA1, HSF1, KLF5, NCOR2, NFKB1, NR3C1, PPARG, RARB, RELA, RUNX1, SMAD3, SMAD4, SMARCA2, SPI1, SRF*
STAT3	*HOXA1, AKT1, BIRC5, CCR5, CD274, CEBPD, CRP, CYP19A1, FAAH, FGG, FOS, HGF, IL10, IRF1, LBP, MMP1, MMP7, MUC1, MYD88, NOS3, REG1A, ROR1, SREBF1, TP53, TP63, VEGFA, VIM*	*APRF, ADMIO1, ADMIO, HIES*	*BIRC5, ESR1, ETS1, FOXA1, IL10, INSM1, IRF1, JM2, NHLH1, NOS3, NR2F1, PBX1, PLAG1, PRO2286, STAT1, TCF3, TEAD1, VDR*	*AR, ATF3, BATF3, CREBBP, GTF2I, HES1, HES5, HESX1, HIC1, HIVEP1, HOXC11, JUN, KLF15, MYOD1, NCOA1, NFKB1, NR3C1, NR4A1, PPARD, RELA, SMARCA4, STAT6, TWIST1, ZFPM2, ZNF281, ZNF557, ZNF829*
**E. coli**				
**Knockdown TF**	**TFs directly connected in regulatory network**	**Aliases (EcoCyc)**	**TFs connected via pathway (EcoCyc)**	
AppY	*DpiA, H-NS*	-	*RpoS, ArcA, H-NS, DpiA*	
ArcA	*Fnr*	*sfrA, cpxC, dye, fexA, msp, seg*	*Fnr*	
Fnr	*ArcA, Fur, IHF*	*nirA, nirR, ossA, oxrA*	*IHF, Fur, ArcA, SoxS*	
OxyR	*CRP*	*momR, mor*	*CRP*	
SoxS	*SoxR, AcrR, Fnr, Fur*	-	*SoxR, MgrR, AcrR, Fnr, Fur*	
